# Ultrasonography and colour Doppler in juvenile idiopathic arthritis: Diagnosis and follow-up of ultrasound-guided steroid injection in the wrist

**DOI:** 10.1186/1546-0096-9-S1-P41

**Published:** 2011-09-14

**Authors:** Louise Laurell, Michel Court-Payen, Susan Nielsen, Marek Zak, Anders Fasth

**Affiliations:** 1 Department of Paediatrics, Skåne University Hospital, Lund University, Sweden; 2 Department of Diagnostic Imaging, Gildhøj Private Hospital, University of Copenhagen, Denmark; 3 Department of Paediatrics, Rigshospital, University of Copenhagen, Denmark; 4 Department of Paediatrics, University of Gothenburg, Sweden

## Background

Wrist inflammation commonly causes growth deformation and is an indicator of poor outcome in JIA [1]. Due to the anatomical complexity of the wrist, defining the exact anatomical location of synovial inflammation and performing palpation-guided injections are difficult.

## Aim

To assess the usefulness of ultrasonography (US) of the wrist for detection of synovial hypertrophy and hyperaemia, as well as for guidance and efficacy evaluation of steroid injections in patients with JIA.

## Methods

15 symptomatic wrists in 11 patients with JIA, 10 girls and 1 boy between 2 and 16 years (median 12.5 years), were assessed clinically and by Doppler-US before and after (1 - 4 weeks) US-guided steroid injection. The presence (no normalization) or absence (normalization) of synovial hypertrophy and Doppler flow (hyperaemia) after treatment was recorded.

## Results

Inflammation was found in all 15 wrists. Table 1-2 show involved anatomical structures and the effects of US-guided steroid injections.

**Table 1:** US diagnosis of synovial hypertrophy and hyperaemia in 15 wrists before injection

Compartment	Number of wrists	Synovial hypertrophy	Hyperaemia
Radio-carpal joint	13	13 (87%)	12 (80%)
Mid-carpal joint	8	8 (53%)	7 (47%)
Tendon sheaths	5	5 (33%)	4 (27%)
All compartments	26	26 (100%)	23 (88%)

**Table 2:** Effect on synovial hypertrophy 1 week and 4 weeks after US-guided steroid injection

Normalization Compartment	Number injected	Result after 1 week Normalization		Result after 4 weeks	
		
		Yes	No	Yes	No
Radio-carpal joints	12	6/12 (50%)	6/12 (50%)	10/12 (83%)	2/12 (17%)
Mid-carpal joints	5	2/5 (40%)	3/5 (60%)	4/5 (80%)	1/5 (20%)
Tendon sheaths	4	4/4 (100%)	0/4 (0%)	4/4 (100%)	0/4 (0%)
All compartments	21	12/21 (57%)	9/21 (43%)	18/21 (86%)	3/21 (14%)

## Conclusion

US enabled exact anatomical location of synovial inflammation, guidance of steroid injections and was valuable for follow-up examinations. Our findings suggest that in children with JIA US should be performed before wrist injections and to guide those procedures.
